# Computerized nailfold video‐capillaroscopy in type 2 diabetes: A cross‐sectional study on 102 outpatients

**DOI:** 10.1111/1753-0407.13442

**Published:** 2023-07-09

**Authors:** Giuseppe Lisco, Vincenzo Triggiani

**Affiliations:** ^1^ Interdisciplinary Department of Medicine, Section of Internal Medicine, Geriatrics, Endocrinology and Rare Diseases, School of Medicine University of Bari Aldo Moro Bari Italy

**Keywords:** cardiovascular disease, chronic renal disease, computerized nailfold video‐capillaroscopy, diabetic retinopathy, erectile dysfunction, type 2 diabetes, 计算机化甲襞视频毛细血管镜, 2型糖尿病, 勃起功能障碍, 糖尿病视网膜病变, 慢性肾病, 心血管疾病

## Abstract

**Background:**

Type 2 diabetes (T2D) is a chronic disease that negatively affects vascular health. A careful assessment of chronic complications, including microcirculation, is mandatory. The computerized nailfold video‐capillaroscopy (CNVC) accurately examines the nailfold microvasculature, but its suitability in T2D is currently under investigation.

**Aims:**

To describe nailfold microvasculature in T2D patients regarding the level of glucose control and chronic microvascular and macrovascular complications.

**Methods:**

This is a cross‐sectional study on 102 consecutive and unselected outpatients with T2D who had undergone CNVC examination. The examination was carried out by using an electronic video‐capillaroscope with 300x magnification. Capillaroscopic appearance and capillary changes were described according to well‐established parameters. Capillaroscopic parameters were compared between patients with poor glucose control (HbA1c ≥7%) and those with better glucose control (HbA1c <7%) and between patients with chronic complications and those without. Chronic complications were deduced from the anamnestic, laboratory, and instrumental data and the five‐item International Index of Erectile Function (IIEF‐5) questionnaire.

**Results:**

Nailfold capillaries in patients with HbA1c ≥7% were thicker (*p* = .019) and longer (*p* = .021) than in those with better glucose control. Ectasias (*p* = .017) and microaneurysms (*p* = .045) were more frequently observed in patients with HbA1c ≥7.0% than those with HbA1c <7.0%. Patients with ED, compared to those without, had a lower frequency of bizarre‐shaped capillaries (*p* = .02). Microaneurysms (*p* = .02) were more frequently described in patients with carotid stenosis (>20%) than those without.

**Conclusion:**

Relevant nailfold microvascular alterations were observed in T2D, most of which were associated with poor glycemic control, ED, and carotid stenosis. Further investigation is needed to recognize the role of CNVC in predicting the onset and evolution of chronic complications and monitoring the effectiveness of antihyperglycemic treatments on microcirculation.

## Graphical abstract

Highlights

- Patients with type 2 diabetes (T2D) are prone to developing chronic diabetes‐related complications at the microvascular and macrovascular levels. The Computerized Nailfold Video‐Capillaroscopy (CNVC) provides specific information on both quantitative and qualitative characteristics of microvasculature at the level of fingers.
- The present study aims to characterize nailfold microvascular changes in T2D patients and better describe specific nailfold patterns related to poor glucose control (ie, HbA1c ≥7%) and chronic complications, such as diabetic retinopathy, chronic kidney impairment, atherosclerosis, established cardiovascular disease, and erectile dysfunction (ED). This cross‐sectional cohort study was conducted on 102 consecutive and unselected outpatients with T2D. Patients underwent a comprehensive assessment of nailfold microvasculature by CNVC.
- Specific patterns of nailfold microvascular alteration are observed in T2D, such as tortuosity, ectasias, microaneurysms, and enlarged capillaries. Poor glycemic control is associated with dilated and longer capillaries and a higher frequency of ectasias and microaneurysms. Men with ED had fewer bizarre‐shaped capillaries (a sign of impaired angiogenesis), and those with clinical signs of atherosclerosis (ie, carotid stenosis >20%) had more microaneurysms. Future studies are needed to understand better the role of the CNVC in identifying at‐risk patients requiring specific therapeutic management to prevent the onset and evolution of severe chronic complications.

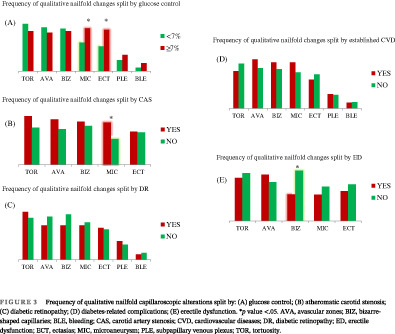

## 摘要

背景: 2型糖尿病（T2D）是一种对血管健康产生负面影响的慢性疾病。对慢性并发症、包括微循环的仔细评估是必须的。计算机化甲襞视频毛细血管镜（CNVC）能够准确检查甲襞微血管，但其是否适合用于T2D尚待研究。本文旨在描述T2D患者的甲襞微血管，探讨血糖控制水平以及慢性微血管和大血管并发症对其的影响。

方法: 这是一项针对102名连续且未经筛选的T2D门诊患者的横断面研究，所有患者均接受CNVC检查。使用电子视频毛细血管镜以300倍的放大率进行检查。根据已确定的参数描述毛细血管镜下外观和毛细血管改变。将毛细血管镜参数在血糖控制不佳（HbA1c≥7%）和血糖控制较好（HbA1c<7%）的患者之间以及有慢性并发症和无慢性并发症的患者之间进行比较。慢性并发症根据病史、实验室和仪器数据以及国际勃起功能指数（IIEF‐5）问卷进行推断。

结果: HbA1c≥7%患者的甲襞毛细血管较厚（p=0.019）且更长（p=0.021），与血糖控制较好的患者相比。HbA1c≥7.0%的患者中毛细血管扩张（p=0.017）和微动脉瘤（p=0.045）的发生率高于HbA1c<7.0%。与无ED患者相比，有ED的患者奇异形状毛细血管的发生率较低（p=0.02）。伴有颈动脉狭窄>20%的患者中微动脉瘤的发生率较高（p=0.02）。

结论: 在2型糖尿病患者中观察到相关的甲襞微血管改变，其中大多数与血糖控制不佳、ED和颈动脉狭窄有关。需要进一步调查以认识到CNVC在预测慢性并发症的发生和进展以及监测抗高血糖药物治疗对微循环的有效性中所起的作用。

## 1 BACKGROUND

People living with type 2 diabetes (T2D) are at risk of developing microvascular and macrovascular complications,^1^ with atherosclerotic cardiovascular disease (CVD) as the most relevant cause of comorbidity and mortality.^2^ Microvascular complications, such as diabetic retinopathy (DR), micro‐ and macroalbuminuria, diabetic neuropathies, and erectile dysfunction, foster the risk of CVD,^3^, ^4^ also affecting the quality of life and general health status.^5^, ^6^

The computerized nailfold video‐capillaroscopy (CNVC) is an imaging technique analyzing the microvasculature at the level of the toes and fingers in feet and hands. The method is used in numerous fields of medicine to assess microvascular injury in several conditions, including non‐rheumatologic disorders such as hypertension,^6^ T2D,^7^ and endocrine diseases.^8^ The CNVC is not currently used in diabetology, even if a previous study found that nailfold examination can easily detect specific capillaroscopic changes in prediabetes and T2D compared to healthy controls and in T2D complicated by diabetic neuropathy.^9^

This study aimed to describe nailfold microvasculature characteristics in patients with T2D, analyzed by the CNVC at the level of nailfold of fingers. In addition, nailfold microvasculature changes were compared according to the level of glucose control and the presence of chronic diabetes‐related complications at the time of the examination.

## 2 MATERIALS AND METHODS

The cross‐sectional study was conducted on 102 consecutive and unselected T2D outpatients who attended the Endocrinology Center of the University of Bari from January to September 2018. Each patient was informed about the study's purposes and provided written informed consent to participate before undergoing the examination.

Inclusion criteria were as follows: established diagnosis of type 2 diabetes and age ≥18 years. Exclusion criteria were caffeine consumption and cigarette smoking within 2 hours before the CNVC examination and confirmed diagnosis of connective tissue disorders.

A single operator (G.L.) performed the CNVC examination. The examination was conducted after proper handwashing with water and soap and a complete ceramic oil immersion of fingers to obtain clear images. Fingers with recent signs of trauma were excluded to avoid false positive results. To prevent significant peripheral vasomotion, patients were examined at comfortable room temperatures between 23° and 25°C.

Nailfold microvasculature was described, and relevant morphological changes were classified according to quantitative and qualitative parameters. The former included capillary density, depth, and length. The latter were tortuosity (TOR), bizarre‐shaped capillaries (BIZ), ectasias (ECT), avascular areas (AVA), microaneurysm (MIC), bleeding (BLE), and visible subpapillary venous plexus (PLE). The criteria used to describe quantitative and qualitative parameters are explained elsewhere.^10^

**FIGURE 1:**
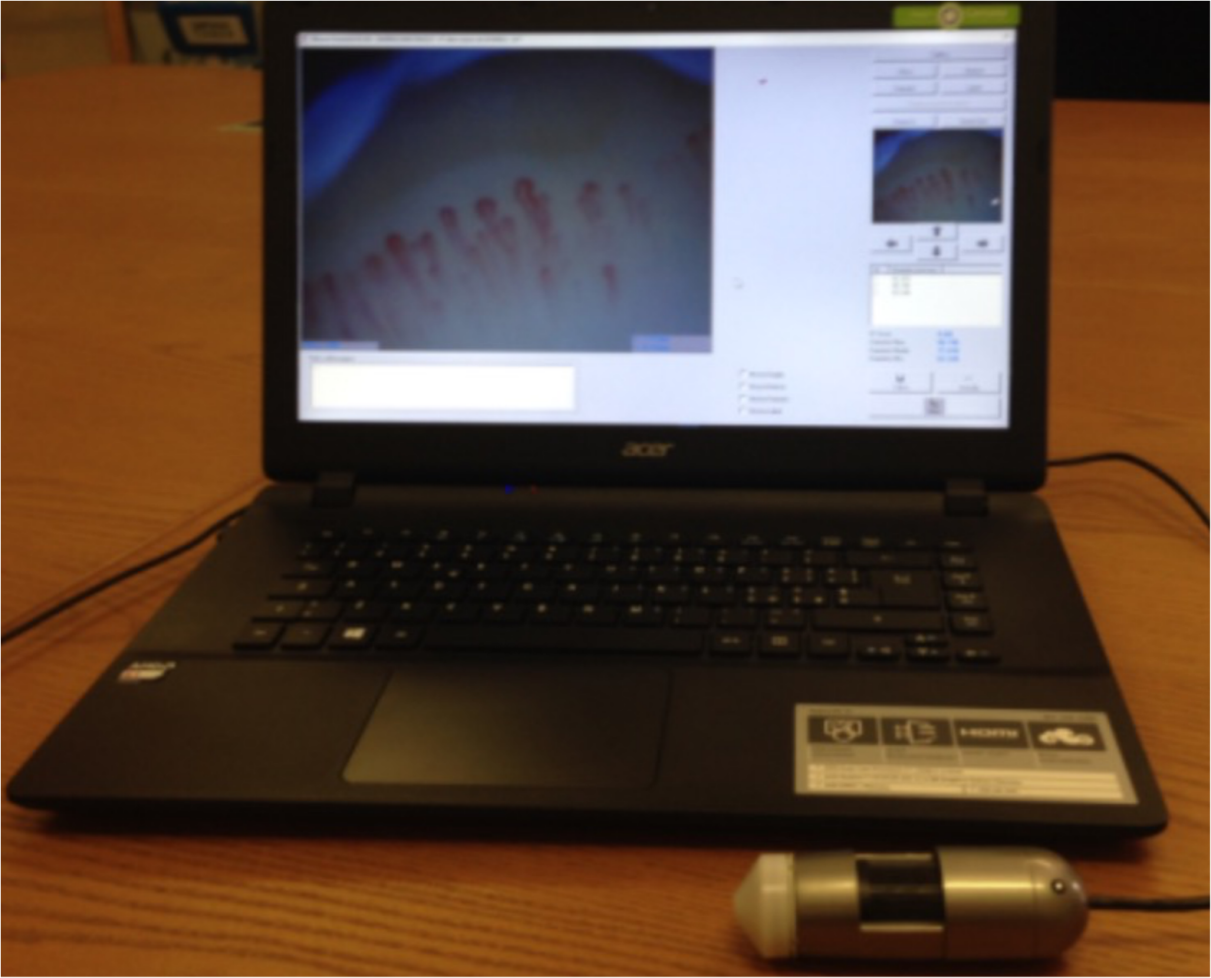
Video‐capillaroscope connected to the software PicocapMicrolab Electronica (Padua). The image displays the nailfold capillary distal row of a 72‐year‐old patient with type 2 diabetes.

**FIGURE 2:**
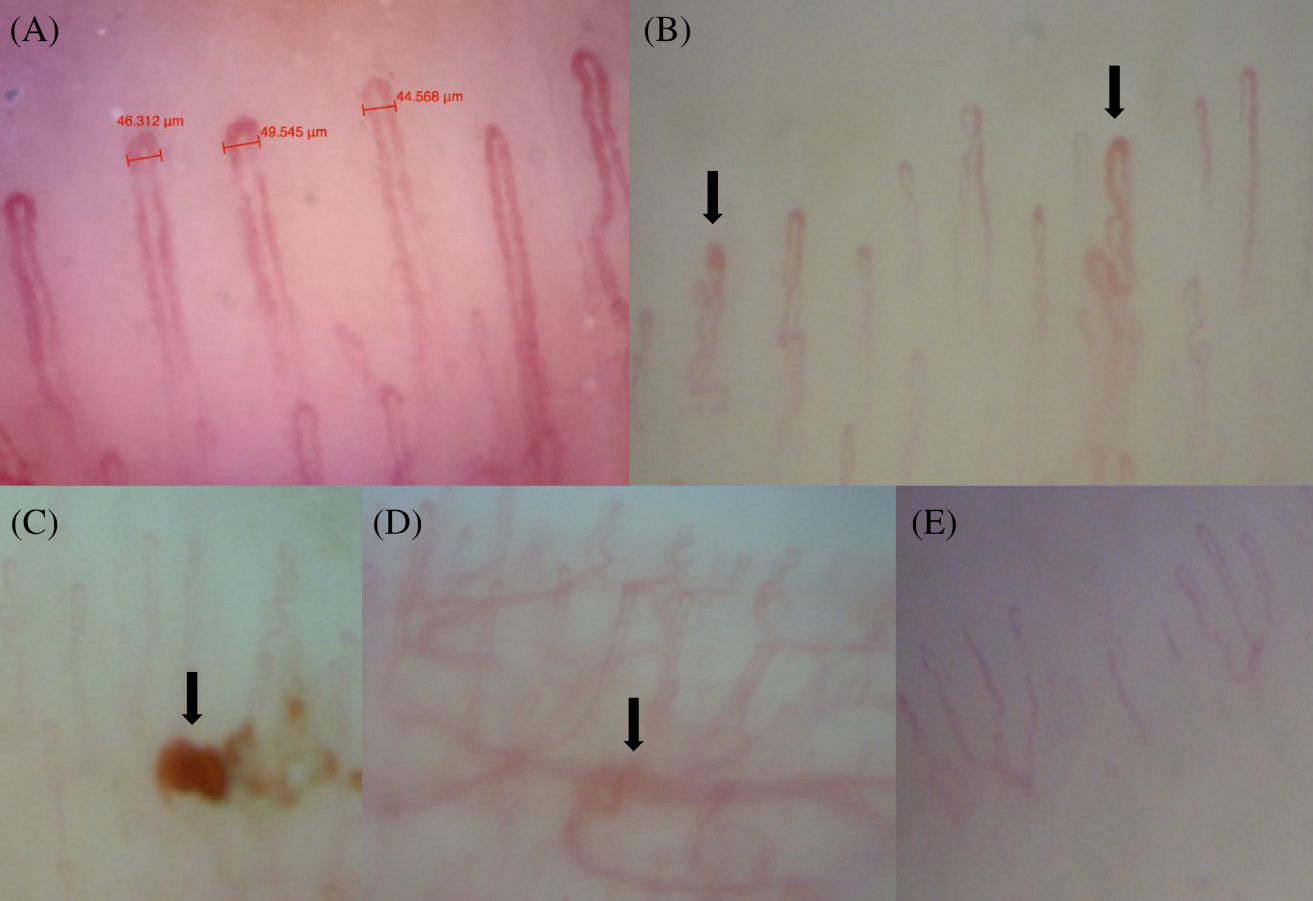
The picture shows some explicative nailfold capillaroscopic patterns of the study population. (A) typical pattern; (B) tortuosity (black arrows); (C) capillary bleeding (black arrow); (D) subpapillary plexus (black arrow); (E) ramified pattern.

The CNVC examination was performed using the PicocapMicrolab Electronica® (Padua), as shown in Figure 1. Nailfold images were collected from the distal row of the fourth and fifth fingers of the nondominant hand. At least three shots were acquired and registered from the nailfold middle in examined fingers (Figure 2). Nailfold characteristics were described in each image, and all findings were shown as an average.

Medical history and physical examination, recent laboratory tests, and instrumental examinations were available for each patient. Information on chronic complications was obtained after a comprehensive appraisal of medical history, laboratory tests (e.g., serum creatinine and urinary albumin‐to‐creatinine ratio), electrocardiogram, carotid ultrasound, and fundoscopic examination. Lastly, the five‐item International Index of Erectile Function (IIEF‐5) questionnaire was administered to men for diagnosing and classifying erectile dysfunction (ED).^11^

The glucose control was defined based on the glycated hemoglobin (HbA1c) levels. More precisely, HbA1c <7% defined patients with optimal glucose control, whereas those having HbA1c ≥7% had worse glucose control.

## 3 STATISTICS

Descriptive data are shown as numbers, percentages, mean (or median), and SD (or quartile range). Comparisons between categorical variables were performed by the chi‐square test. A two‐tailed *t* test was performed to evaluate mean differences between the two groups in the case of continuous variables with a normal distribution (assessed by the Shapiro–Wilk test). A *p* value ≤.05 was considered statistically significant. Statistical analyses were carried out with R.

## 4 RESULTS

### 4.1 Baseline characteristics

The cross‐sectional study included 102 outpatients (68 men and 34 women). Ten patients were excluded due to signs of trauma at the level of the fourth and fifth fingers of both hands (two), evidence of heavy manual works significantly affecting the nailfold microvasculature (two), having consumed caffeine or cigarette smoke before the examination (five), and suspicion of the Raynaud phenomenon (one).

Arterial hypertension (74%) and hypercholesterolemia (72%) were the main comorbidities in the study population. Around 80% of the entire cohort exhibited a weight excess. Thirty‐seven patients (36.2%) were overweight (body mass index [BMI]: 25–29.9 kg/m^2^), and 45 (44.1%) were obese, defended as BMI ≥30 kg/m^2^. Sixteen individuals (15.6%) were current smokers (12 men; 4 women), and 31 (30.3%) smoked in the past (29 men; 2 women).

Mean age (men: 62.9 [10.8] years vs. women: 64.2 [12] years, *p* = .2), and the evolution of T2D (men 12.3 [9.2] years vs. women 11.8 [9] years, *p* = .5), arterial hypertension (men: 10.8 [7.9] years vs. women: 14 [8.7] years, *p* = .06), and hypercholesterolemia (men: 7.8 [7] years vs. women: 9.8 [9.6] years, *p* = .08) were not statistically different between the two genders.

### 4.2 Biochemical and anthropometric parameters

Table 1 shows the leading laboratory and anthropometric parameters of the study population. The mean value of HbA1c was 7.0%. Slightly more than half of patients (51.7%) achieved an adequate glucose control. Glucose control was similar between men and women. Low‐density lipoprotein cholesterol levels were lower in men than women (men: 81.4 [31.4] mg/dL vs. women: 99.1 [41.7] mg/dL, *p* = .02). Non‐high‐density lipoprotein (HDL) cholesterol levels were lower in men than women (men: 106.5 [34.7] mg/dL vs. women: 124.7 [44.3] mg/dL, *p* = .03). Conversely, HDL cholesterol levels were higher in women than men (women: 54.3 [12.8] mg/dL vs. men: 46.3 [11.6] mg/dL, *p* = .002).

**TABLE 1:** Laboratory and anthropometric parameters of the study population are split by gender (men: 68; women: 38).

Variable	Mean (SD)	UM	*p* value
FPG	126.2 (32.9)	mg/dL	.08
Women	117.7 (32.6)		
Men	129.9 (32.7)		
HbA1c	7.0 (1)	%	.06
Women	6.7 (1.1)		
Men	7.2 (0.9)		
Non‐HDL cholesterol	109.5 (41.9)	mg/dL	.03*
Women	124.7 (44.3)		
Men	106.5 (34.7)		
HDL cholesterol	49.6 (13.2)	mg/dL	.002*
Women	54.3 (12.8)		
Men	46.3 (11.6)		
LDL cholesterol	87.1 (35.7)	mg/dL	.02*
Women	99.1 (41.7)		
Men	81.4 (31.4)		
Triglycerides	132.2 (85.2)	mg/dL	.8
Women	133.7 (95.9)		
Men	130.1 (80.4)		
eGFR	84.1 (19.5)	mL/min/1.73 m^2^	.4
Women	86.4 (17.6)		
Men	83 (20.4)		
ACR	56.9 (150)	mg/g	.97
Women	56 (175.5)		
Men	57.3 (139.3)		
BMI	31.4 (2.4)	kg/m^2^	.4
Women	31.7 (2.7)		
Men	29.8 (2.2)		
WC	123.2 (12.3)	cm	.6
Women	121.9 (14.5)		
Men	124.5 (11.1)		

Abbreviations: ACR, albumin to creatinine ratio; BMI, body mass index; eGFR, estimated filtration rate; FPG, fasting plasma glucose; HbA1c, glycated hemoglobin; HDL, high density lipoproteins; LDL, low density lipoproteins; UM, unit of measurement; WC, waist circumference.

* Statistically significant (*p* value <.05).

The BMI and waist circumference were not significantly different between the two genders.

### 4.3 Diabetes‐related complications

Patients with renal impairment were defined as those having a stable estimated glomerular filtration rate <60 mL/min/1.73 m^2^ or urine albumin to creatinine ratio >30 mg/g. They represented 32% of the study population (33 individuals).

DR has been diagnosed in 31 patients (30.5%), and 24 (23.5%) had established CVD (composite of acute coronary syndromes, coronary or carotid artery revascularization, stroke, and peripheral artery occlusion or revascularization).

Carotid intima‐media thickness ranged from 0.6 to 1.4 mm (mean 0.91 [0.18] mm). An atherosclerotic stenosis in the carotid artery (CAS) was seen in 60 patients (58%), 45 men (59.4%), and 15 women (47%).

Clinically relevant erectile dysfunction (IIEF‐5 < 17 points) was diagnosed in 20 men (19.6%).

### 4.4 CNVC findings

Capillary findings were defined as summarized in Table 2. They were split into two main categories, quantitative and qualitative parameters, according to Tavakol et al.^12^ The capillary density was 7.7 (1.6) per mm^2^ with no difference in men compared with women. Capillary depths and lengths were 42.2 (9.9) μm and 193.5 (91.3) μm, respectively. Although nailfold capillaries were longer and deeper in men than women, this difference was not statistically significant.

**TABLE 2:** CNVC parameters collected and analyzed in the study population.^10^

Nailfold capillaroscopic parameters	
Quantitative	Qualitative
Capillary density: number of capillary loops observed in the nailfold distal row per mm^2^.	Tortuosity (TOR): capillary with waved, cross‐linked, sinuous or twisted shape in at least 50% of the nailfold distal row per mm^2^.
Capillary diameters (depth and length): number of capillary loops observed per mm^2^ of the visual field (nailfold distal row).	Ectasias (ECT): capillary with apex diameter >50 μm and/or with one or both loop branches thickness >20 μm.
	Capillaries with bizarre shape (BIZ): capillary with highly ramified shape, or shape resembling part of a tree or an animal.
	Avascular areas (AVA): capillary density <7 capillaries/mm^2^ or the absence of two or more consecutive distal row capillaries or capillary loss within a space >500 μm.
	Microaneurysm (MIC): dilatation of the capillary wall at the level of the capillary loop apex and/or one or both loop branches.
	Bleeding (BLE): hemosiderin deposits.
	Visible subpapillary venous plexus (PLE).

Abbreviation: CNVC, computerized nailfold video‐capillaroscopy.

The main qualitative findings observed in the study population were as follow: TOR (65.7%), AVA and BIZ (62.7%), MIC (53.9%), ECT (49%), PLE (20.6%), and BLE (8.8%). The frequency of these qualitative parameters was similar in men and women (Table 3).

**TABLE 3:** CNVC qualitative parameters of the study population are split by gender.

Variables	Mean (SD)	*p* value
TOR	67/102 (65.7)	.38
Women	20/34 (58.8)	
Men	47/68 (69.1)	
AVA	64/102 (62.7)	.052
Women	26/34 (70.6)	
Men	38/68 (55.9)	
BIZ	64/102 (62.7)	.52
Women	23/34 (67.6)	
Men	41/68 (60.3)	
MIC	55/102 (53.9)	.29
Women	21/34 (61.7)	
Men	34/68 (50)	
ECT	50/102 (49)	.53
Women	15/34 (44.1)	
Men	35/68 (51.6)	
PLE	21/102 (20.6)	1
Women	7/34 (20.6)	
Men	14/68 (20.6)	
BLE	84.1 (8.8)	1
Women	3/34 (8.8)	
Men	6/68 (8.8)	

Abbreviations: AVA, avascular zones; BIZ, bizarre‐shaped capillaries; BLE, bleeding; CNVC, computerized nailfold video‐capillaroscopy; ECT, ectasias; MIC, microaneurysm; PLE, subpapillary venous plexus; TOR, tortuosity.

### 4.5 CNVC findings and the level of glucose control

Patients with worse glucose control (HbA1c ≥7.0%) showed thicker nailfold capillaries (42.8 [9.8] vs. 38.2 [9.4] μm; *p* = .019) compared to those who had achieved better glucose control (HbA1c <7.0%). Shorter nailfold capillaries (174.8 [73.4] vs. 216.4 [103.8] μm, *p* = .021) were observed in patients with better glucose control than those with worse glucose control.

A statistically significant higher frequency of ECT (62.5% vs. 37.7%, *p* = .017) and MIC (64.6% vs. 43.4%, *p* = .045) was found in patients with worse glucose control than those with HbA1c <7.0%.

The remaining qualitative CNVC parameters were similar between the two groups.

### 4.6 CNVC findings and chronic diabetic complications

Patients with chronic renal impairment displayed slightly longer capillaries than those without renal impairment (219.6 [91.4] vs. 184.7 [89.9] μm), but the difference was not statistically significant (*p* = .07). Moreover, a lower frequency of BIZ was found in patients with ED (IIEF‐5 < 17 points) compared to those without (35% vs. 68.9%, *p* = .02).

**FIGURE 3:**
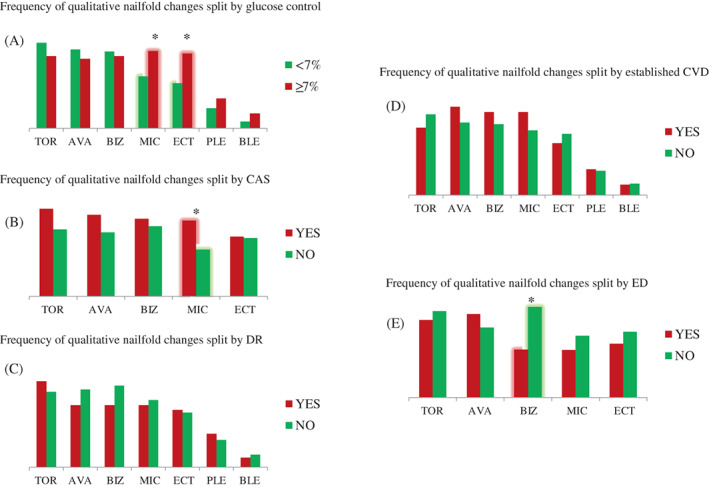
Frequency of qualitative nailfold capillaroscopic alterations split by: (A) glucose control; (B) atheromatic carotid stenosis; (C) diabetic retinopathy; (D) diabetes‐related complications; (E) erectile dysfunction. *p value <.05. Abbreviations: AVA, avascular zones; BIZ, bizarre‐shaped capillaries; BLE, bleeding; CAS, carotid artery stenosis; CVD, cardiovascular diseases; DR, diabetic retinopathy; ED, erectile dysfunction; ECT, ectasias; MIC, microaneurysm; PLE, subpapillary venous plexus; TOR, tortuosity.

Patients with CAS >20% compared to those without CAS had a higher frequency of MIC (63.3% vs. 39%, p = .02). No difference in the frequency of qualitative parameters was found in patients with DR than those without DR and in patients with established CVD than those without CVD (Figure 3).

## 5 DISCUSSION

The CNVC is a noninvasive and easy‐to‐use diagnostic tool that provides real‐time information on the general microvascular architecture and specific details of small nailfold vessels. Accurate nailfold examination should include the capillary density, shape, and distribution. In healthy individuals, different capillaroscopic patterns were described so that a “physiologic” nailfold appearance is challenging to be well‐defined. Three healthy capillaroscopic patterns have been identified,^13^ including the following: (a) the “normal pattern” made up of 2–5 “U‐shaped” loops and ≤2 TOR per mm^2^; (b) the “perfect normal pattern” composed of more than five “U shaped” loops per mm^2^; and (c) the “unusual normal pattern,” which includes at least one “bushy” loop, one microhemorrhage, or more than four “crossed” loops per mm^2^. Nevertheless, several factors may affect the nailfold microvasculature appearance including age, gender, and lifestyle habits (eg, cigarette smoking, heavy physical exercise). Therefore, a high variability in healthy and pathological findings could be expected.^14^

However, the CVNC examination provides valuable information about the type and severity of microvascular involvement in chronic diseases and for evaluating the efficacy of treatments. The CNVC is used to assess scleroderma spectrum disorders. In patients with systemic sclerosis, capillary abnormalities appear and evolve in a clearly defined sequence called the “scleroderma pattern,” which strongly correlates with splanchnic involvement. The CNVC findings are currently listed as one of the leading criteria to stage the severity of systemic sclerosis, according to the European League Against Rheumatism.^15^

Morphological remodeling of the capillary wall occurs due to specific connective damage and may also result from hydrodynamic and metabolic disorders, including T2D. Microvascular dysfunction is strictly related to hyperglycemia, and a reciprocal relationship between the two phenomena is also known.^16^ Patients with arterial hypertension and diabetes^17^ exhibit a microvascular imbalance at the level of the precapillary sphincters, leading to raised hydrostatic pressure and consequent enlargement and twisting of the capillary axis. This pathophysiological phenomenon generates crossing capillaries, one of the first signs of capillary adaptation. Another study confirmed an elevated frequency of branched/ramified capillaries and TOR in T2D.^18^ As recently reviewed by Maldonado et al,^19^ unusual nailfold capillaroscopic patterns (such as TOR, ECT, AVA, and BIZ) can also be identified in individuals with diabetes mellitus. Loss of pericytes and capillary wall enlargement are two common findings in T2D, and they lead to ECT, MIC, and BLE due to pathological increase of vascular permeability. These alterations are similar to those described in diabetes‐related retinal injury.^20^

Extensive chronic hypoxic injury conducts to either neoangiogenesis or capillary rarefaction. The presence of nailfold PLE and BIZ is frequently described in some disorders, including T2D. PLE and BIZ result from newborn vessels developing and chaotic neoangiogenesis.^21^ AVA results from a capillary desertification process due to severe and extensive tissue hypoperfusion/hypoxia and insufficient neoangiogenesis restoring stimuli.

This cross‐sectional study analyzed nailfold microvascular architecture and capillary details in T2D patients. Over half of the participants achieved optimal glucose control (HbA1c <7%). CAS was the most relevant chronic complication diagnosed in the study population, especially men (60%). Data are consistent with results previously described in T2D.^22^ The frequencies of both chronic renal impairment and DR in this study were similar to those observed by other authors (35% and 30%, respectively).^23^ CVD and ED were diagnosed in fewer patients (around 20%).^24^

The CNVC assessment showed an elevated frequency of TOR (66%), AVA (63%), BIZ (63%), MIC (54%), and ECT (49%). These findings were comparable to those observed in an Ecuadorian–Hispanic population with parallel mean age (57 [8.9] years) and evolution of T2D (around 12 years). Nailfold microvascular abnormalities were observed in 83% of the population, and TOR (63%) represented the main capillary alteration.^25^ However, in our study, AVA and ECT were slightly more frequent than those found by Maldonado.

Patients with worse glucose control during the examination displayed thicker and longer capillaries and a higher frequency of MIC and ECT compared to those with better control. These data support the hypothesis that glucose control could negatively affect microvasculature health at the level of the fingers.^26^

The so‐called “diabetic nailfold capillaropathy”^27^ is the local expression of a systemic microvascular disorder that is characterized by pericyte damage, capillary wall enlargement and elongation, and variable structural changes, including TOR, ECT, AVA, and MIC.^28^ This phenomenon could be particularly more evident in patients with worse glucose control. Given the same pathogenetic hypothesis, a higher frequency of detrimental nailfold patterns should be expected in patients with chronic diabetes‐related complications. Uyar et al^29^ found a high prevalence of TOR, ECT, and “bushy” capillaries in T2D patients with DR. The reduction of capillary length, irregular distribution, and abnormal capillaries morphology were more frequently observed in patients with prediabetes and T2D than in healthy controls, as well as in T2D patients with neuropathy compared to those without.^9^ Previous findings in type 1 and T2D^30^ also indicated a direct relationship between the severity of the retinal vascular injury and several nailfold microvasculature signs of adaptation such as ECT, MIC, and TOR. Lastly, the capillary rarefaction at rest and after brachial artery ischemia was directly associated with the level of urinary albumin excretion, resulting in an independent risk factor of the pathogenesis of microalbuminuria.^31^ Our data did not find a significantly higher number of capillaroscopic alterations in patients with DR and renal impairment. However, patients with ED had more AVA and fewer BIZ than those without. These findings could reflect the presence of a chronic hypoxic injury as a common pathophysiological mechanism explaining ED and finger nailfold microvascular deterioration.

Irrespective of T2D, some authors^32^ evaluated the relationship between nailfold microvasculature patterns (or digital flowmetry) and the severity of ED in patients with systemic sclerosis. Despite controversial results, AVA and ECT and a relevant reduction in the digital flowmetry were found in the nailfold and penile vessels of men with ED. Specific data on the relationship between nailfold microvascular patterns and penile microvascular damage in T2D are lacking, and further investigation is required to clarify this issue.

This study has some strengths and limitations. First, the cross‐sectional design of this study provides only epidemiological information on a small sample of T2D individuals. Second, the CNVC examination in healthy and unhealthy subjects is affected by a relevant heterogenicity in terms of the morphological appearance of nailfold microvasculature. Moreover, the CNVC assessment significantly depends on the operator's expertise, and both may affect the accuracy when defining the architecture of nailfold microvasculature. In this study, a single‐operator examination was carried out according to standardized methods before, during, and after the examination to improve the quality of nailfold images and interpretation of results (e.g., finger selection, room temperature, exposure to interfering substances such as caffeine and cigarette smoking, adequate magnification, number of captured images, selection of appropriate shots). Furthermore, the single‐operator examination avoided an additional interoperator bias in describing nailfold appearance.

Automated and semiautomated systems are operated to reduce the intra‐ and interoperator variability of microvasculature analyses. Thanks to movement correction, selection of the frame range and positioning of the region of interest, automatic detection of capillaries, and manual correction of detected capillaries, automated algorithms,^33^ and machine learning significantly improve the accuracy, reproducibly, and standardization of CNVC results, also predicting diabetes‐related outcomes.^34^

Although not yet standardized, the CVNC examination in patients with diabetes mellitus may provide further information on the microvascular injury in T2D. More research is needed to characterize better the diabetes‐related nailfold microvasculature damage and its relationship with other chronic complications, such as CVD and high cardiovascular risk. For example, identifying morphological or functional capillaroscopic alterations potentially associated with silent coronary artery disease could implement current algorithms to identify patients at high risk of asymptomatic coronary artery disease. In addition, nailfold examination could be used to assess quickly and monitor the progression of complications over time and the response to antihyperglycemic treatments.

## 6 CONCLUSION

CNVC is an easy, inexpensive, and valuable tool to evaluate the microvasculature damage at the level of the fingers in T2D. Further studies are urgently needed to explore better the role of nailfold examination in improving the management of the disease over time. The current limitation is the interpretation of results, which could be easily overcome by using newer computerized systems to improve the automatic assessment and understanding of nailfold images.

## AUTHOR CONTRIBUTIONS

Giuseppe Lisco conceived the study, collected and analyzed data, developed the database, provided statistical expertise, and drafted the manuscript. Vincenzo Triggiani read the text, provided feedback, and approved the final version.

## FUNDING INFORMATION

None to declare.

## CONFLICT OF INTEREST STATEMENT

There are neither competing conflicts of interest in connection with the submitted article nor industry relationship; thus, the authors deny any competing financial claim related to this study.

## References

[1] Shah AD , Langenberg C , Rapsomaniki E , et al. Type 2 diabetes and incidence of cardiovascular diseases: a cohort study in 1·9 million people. Lancet Diabetes Endocrinol. 2015;3(2):105‐113. doi:10.1016/S2213-8587(14)70219-0 25466521 PMC4303913

[2] Laakso M . Heart in diabetes: a microvascular disease. Diabetes Care. 2011;34(Suppl 2):S145‐S149. doi:10.2337/dc11-s209 21525446 PMC3632152

[3] Hsu CY , Lee CM , Chou KY , et al. The Association of Diabetic Retinopathy and Cardiovascular Disease: a 13‐year Nationwide population‐based cohort study. Int J Environ Res Public Health. 2021;18(15):8106. doi:10.3390/ijerph18158106 34360398 PMC8345672

[4] Stehouwer CD , Smulders YM . Microalbuminuria and risk for cardiovascular disease: analysis of potential mechanisms. J Am Soc Nephrol. 2006;17(8):2106‐2111. doi:10.1681/ASN.2005121288 16825333

[5] Lisco G , Triggiani V , Bartolomeo N , et al. The role of male hypogonadism, aging, and chronic diseases in characterizing adult and elderly men with erectile dysfunction: a cross‐sectional study. Basic Clin Androl. 2023;33(1):5. doi:10.1186/s12610-022-00182-8 37020191 PMC10077617

[6] Pretolani E , Salvi P , Montaguti L , Pretolani M . Modificazioni emoreologiche e microcircolatorie nell'iperteso [the blood rheological and microcirculatory changes in the hypertensive patient]. Cardiologia. 1991;36(12 Suppl 1):355‐363. 1841791

[7] Halfoun VL , Pires ML , Fernandes TJ , Victer F , Rodrigues KK , Tavares R . Videocapillaroscopy and diabetes mellitus: area of transverse segment in nailfold capillar loops reflects vascular reactivity. Diabetes Res Clin Pract. 2003;61(3):155‐160. doi:10.1016/s0168-8227(03)00111-6 12965104

[8] Schiavon F , Maffei P , Martini C , et al. Morphologic study of microcirculation in acromegaly by capillaroscopy. J Clin Endocrinol Metab. 1999;84(9):3151‐3155. doi:10.1210/jcem.84.9.5952 10487679

[9] Hsu PC , Liao PY , Chang HH , Chiang JY , Huang YC , Lo LC . Nailfold capillary abnormalities are associated with type 2 diabetes progression and correlated with peripheral neuropathy. Med (Baltimore). 2016;95(52):e5714. doi:10.1097/MD.0000000000005714 PMC5207569 28033273

[10] Lisco G , Cicco G , Cignarelli A , Garruti G , Laviola L , Giorgino F . Computerized video‐Capillaroscopy alteration related to diabetes mellitus and its complications. Adv Exp Med Biol. 2018;1072:363‐368. doi:10.1007/978-3-319-91287-5_58 30178372

[11] Rosen RC , Cappelleri JC , Smith MD , Lipsky J , Peña BM . Development and evaluation of an abridged, 5‐item version of the International Index of Erectile Function (IIEF‐5) as a diagnostic tool for erectile dysfunction. Int J Impot Res. 1999;11(6):319‐326. doi:10.1038/sj.ijir.3900472 10637462

[12] Etehad Tavakol M , Fatemi A , Karbalaie A , Emrani Z , Erlandsson BE . Nailfold Capillaroscopy in rheumatic diseases: which parameters should Be evaluated? Biomed Res Int. 2015;2015:974530. doi:10.1155/2015/974530 26421308 PMC4569783

[13] Ingegnoli F , Gualtierotti R , Lubatti C , et al. Nailfold capillary patterns in healthy subjects: a real issue in capillaroscopy. Microvasc Res. 2013;90:90‐95. doi:10.1016/j.mvr.2013.07.001 23880032

[14] Nakajima T , Nakano S , Kikuchi A , Matsunaga YT . Nailfold capillary patterns correlate with age, gender, lifestyle habits, and fingertip temperature. PLoS One. 2022;17(6):e0269661. doi:10.1371/journal.pone.0269661 35704663 PMC9200324

[15] Smith V , Thevissen K , Trombetta AC , et al. Nailfold Capillaroscopy and clinical applications in systemic sclerosis. Microcirculation. 2016;23(5):364‐372. doi:10.1111/micc.12281 27086648

[16] Stehouwer CDA . Microvascular dysfunction and hyperglycemia: a vicious cycle with widespread consequences. Diabetes. 2018;67(9):1729‐1741. doi:10.2337/dbi17-0044 30135134

[17] Han HC . Twisted blood vessels: symptoms, etiology and biomechanical mechanisms. J Vasc Res. 2012;49(3):185‐197. doi:10.1159/000335123 22433458 PMC3369246

[18] Bakirci S , Celik E , Acikgoz SB , et al. The evaluation of nailfold videocapillaroscopy findings in patients with type 2 diabetes with and without diabetic retinopathy. North Clin Istanb. 2018;6(2):146‐150. doi:10.14744/nci.2018.02222 31297481 PMC6593916

[19] Maldonado G , Guerrero R , Paredes C , Rios C . Nailfold Capillaroscopy a non‐invasive tool for direct observation of microvascular damage in diabetes mellitus: review. JSM Atheroscler. 2017;2(4):1037.

[20] Beltramo E , Porta M . Pericyte loss in diabetic retinopathy: mechanisms and consequences. Curr Med Chem. 2013;20(26):3218‐3225. doi:10.2174/09298673113209990022 23745544

[21] Cicco G , Cicco S . Hemorheology and microcirculation in some pathologies of internal medicine. Minerva Med. 2007;98:625‐631. 18299675

[22] De Angelis M , Scrucca L , Leandri M , et al. Prevalence of carotid stenosis in type 2 diabetic patients asymptomatic for cerebrovascular disease. Diabetes Nutr Metab. 2003;16(1):48‐55. 12848305

[23] Pugliese G , Solini A , Bonora E , et al. Chronic kidney disease in type 2 diabetes: lessons from the renal insufficiency and cardiovascular events (RIACE) Italian multicentre study. Nutr Metab Cardiovasc Dis. 2014;24(8):815‐822. doi:10.1016/j.numecd.2014.02.013 24780515

[24] Derosa G , Romano D , Tinelli C , D'Angelo A , Maffioli P . Prevalence and associations of erectile dysfunction in a sample of Italian males with type 2 diabetes. Diabetes Res Clin Pract. 2015;108(2):329‐335. doi:10.1016/j.diabres.2015.01.037 25747572

[25] Maldonado G , Guerrero R , Paredes C , Ríos C . Nailfold capillaroscopy in diabetes mellitus. Microvasc Res. 2017;112:41‐46. doi:10.1016/j.mvr.2017.03.001 28274735

[26] Pazos‐Moura CC , Moura EG , Bouskela E , Torres‐Filho IP , Breitenbach MM . Nailfold capillaroscopy in diabetes mellitus: morphological abnormalities and relationship with microangiopathy. Braz J Med Biol Res. 1987;20(6):777‐780. 3455257

[27] Barchetta I , Riccieri V , Vasile M , et al. High prevalence of capillary abnormalities in patients with diabetes and association with retinopathy. Diabet Med. 2011;28(9):1039‐1044. doi:10.1111/j.1464-5491.2011.03325.x 21517956

[28] Ciaffi J , Ajasllari N , Mancarella L , Brusi V , Meliconi R , Ursini F . Nailfold capillaroscopy in common non‐rheumatic conditions: a systematic review and applications for clinical practice. Microvasc Res. 2020;131:104036. doi:10.1016/j.mvr.2020.104036 32603698

[29] Uyar S , Balkarlı A , Erol MK , et al. Assessment of the relationship between diabetic retinopathy and Nailfold capillaries in type 2 diabetics with a noninvasive method: Nailfold Videocapillaroscopy. J Diabetes Res. 2016;2016:7592402. doi:10.1155/2016/7592402 28058264 PMC5187472

[30] Martens RJ , Henry RM , Houben AJ , et al. Capillary rarefaction associates with albuminuria: the Maastricht study. J Am Soc Nephrol. 2016;27(12):3748‐3757. doi:10.1681/ASN.2015111219 27160406 PMC5118486

[31] Rosato E , Barbano B , Gigante A , et al. Erectile dysfunction, endothelium dysfunction, and microvascular damage in patients with systemic sclerosis. J Sex Med. 2013;10(5):1380‐1388. doi:10.1111/jsm.12110 23444914

[32] Berks M , Tresadern P , Dinsdale G , et al. An automated system for detecting and measuring nailfold capillaries. Med Image Comput Comput Assist Interv. 2014;17(Pt 1):658‐665. doi:10.1007/978-3-319-10404-1_82 25333175 PMC4936512

[33] Cutolo M , Trombetta AC , Melsens K , et al. Automated assessment of absolute nailfold capillary number on videocapillaroscopic images: proof of principle and validation in systemic sclerosis. Microcirculation. 2018;25(4):e12447. doi:10.1111/micc.12447 29527781

[34] Shah R , Petch J , Nelson W , et al. Nailfold capillaroscopy and deep learning in diabetes. J Diabetes. 2023;15(2):145‐151. doi:10.1111/1753-0407.13354 36641812 PMC9934957

